# Clinical Sepsis Phenotypes in Critically Ill Patients

**DOI:** 10.3390/microorganisms11092165

**Published:** 2023-08-27

**Authors:** Georgios Papathanakos, Ioannis Andrianopoulos, Menelaos Xenikakis, Athanasios Papathanasiou, Despoina Koulenti, Stijn Blot, Vasilios Koulouras

**Affiliations:** 1 Department of Intensive Care Medicine, University Hospital of Ioannina, 45500 Ioannina, Greece; jandri0@yahoo.gr (I.A.); mxenikakis@yahoo.gr (M.X.); thanasis.papathanasiou@gmail.com (A.P.); vpkoulouras@yahoo.gr (V.K.); 2 UQ Centre for Clinical Research, Faculty of Medicine, The University of Queensland, Brisbane, QL 4029, Australia; d.koulenti@uq.edu.au; 3 Second Critical Care Department, Attikon University Hospital, Rimini Street, 12462 Athens, Greece; 4 Department of Internal Medicine & Pediatrics, Ghent University, 9000 Ghent, Belgium; stijn.blot@ugent.be

**Keywords:** sepsis, heterogeneity, phenotype, machine learning, precision medicine

## Abstract

Sepsis, defined as the life-threatening dysregulated host response to an infection leading to organ dysfunction, is considered as one of the leading causes of mortality worldwide, especially in intensive care units (ICU). Moreover, sepsis remains an enigmatic clinical syndrome, with complex pathophysiology incompletely understood and a great heterogeneity both in terms of clinical expression, patient response to currently available therapeutic interventions and outcomes. This heterogeneity proves to be a major obstacle in our quest to deliver improved treatment in septic critical care patients; thus, identification of clinical phenotypes is absolutely necessary. Although this might be seen as an extremely difficult task, nowadays, artificial intelligence and machine learning techniques can be recruited to quantify similarities between individuals within sepsis population and differentiate them into distinct phenotypes regarding not only temperature, hemodynamics or type of organ dysfunction, but also fluid status/responsiveness, trajectories in ICU and outcome. Hopefully, we will eventually manage to determine both the subgroup of septic patients that will benefit from a therapeutic intervention and the correct timing of applying the intervention during the disease process.

## 1. Introduction

Ancient Greeks used the word “sepsis” (σήψη) to describe putrefaction. Initially, sepsis was used to depict the consequences of a process, and not the cause [1]. It was not until the 19th century and the identification of microorganisms as the cause of infection that the term ‘sepsis’ was used to describe a clinical disease caused by severe infection. Currently, sepsis is defined as the life-threatening dysregulated host response to an infection leading to dysfunction of multiple organs [2]. Notwithstanding important medical advances, sepsis even now poses a major challenge for both clinicians and trialists, with its treatment still limited to antibiotics, fluid therapy and organ supportive therapy. As for many years no new therapies have become available, it is still very difficult to deal with and continues to be a leading cause of mortality worldwide, affecting both resource-rich and resource-restricted countries [3,4] and taking its toll across all age groups [5,6,7,8,9,10,11]. A recent study estimated that in 2017 sepsis occurred in 48.9 million people globally, and was responsible for 11 million deaths [12]. Thus, the World Health Organization declared sepsis as a global health priority [13].

In intensive care units (ICUs), in particular, regardless of whether due to community- or healthcare-associated infections, sepsis also remains the leading cause of death [14,15,16]. Although intensive care medicine societies developed consensus definitions for sepsis and septic shock aiming towards the harmonization of diagnosis, treatment and clinical trials’ inclusion criteria, the mortality outcomes of different studies still present a wide variability. More specifically, in a meta-analysis of observational studies, septic shock mortality was reported at 46%; however, two important clinical trials of patients with septic shock, conducted almost during the same period, reported control-group mortalities which were extremely contrasting, i.e., 18.8% versus 80% [2,17,18,19,20,21]. On the other hand, in a systematic analysis of 65 septic shock randomized control trials (RCTs) published between 2006 and 2018, the mean mortality rate of the control-groups was 38.6%, with a very wide variability being present (estimated 95%, prediction intervals 13.5–71.7%) [22]. The above may be, at least partially, explained by significant heterogeneity between the clinical studies. In order for the findings to be reproducible and able to be extrapolated, a common phenomenological structure is required in terms of diagnostic and inclusion criteria, case-mix and patient-characteristics. However, in the case of sepsis, this is not possible because of significant intrinsic and extrinsic underlying heterogeneity [23].

If the heterogeneous group of patients with sepsis can be separated into distinct phenotypes, and these phenotypes are characterized by discrete pathophysiology, then they may also differ in responsiveness to therapies; consequently, future therapeutic trials in sepsis could use phenotype assignment to stratify sepsis patients in treatment trials and develop treatment strategies more precisely targeted to specific sepsis phenotypes. The main objective of this review is to provide an overview of different clinical phenotypes existing in critically ill patients with sepsis. A narrative review was considered the most appropriate approach. A literature search was conducted in the bibliographic database PubMed from inception to May 2023; search terms related to ‘sepsis phenotyping’ were combined with terms regarding ‘critical care’. Two authors searched the database independently. A third reviewer mediated any disagreements in the results of the two screeners. Peer-reviewed, published literature, including narrative review papers, were eligible for inclusion. Additional articles were included based on expert opinion. Eventually, studies published in English language that employed data on sepsis phenotyping in critically ill patients were considered for this review.

## 2. Sepsis: A Very Heterogeneous Disease

### 2.1. Patient-Specific Causes of Heterogeneity

Sepsis does not progress or manifest similarly in all patients due to significant heterogeneity between individuals, pathophysiological differences and immunological responses [24,25].

Indicative, but not exclusive, causes of heterogeneity include specific patient characteristics such as age, sex, underlying comorbidities, obesity, alcohol consumption, smoking, medications, nutritional status, the source of the infection, the nature of the infectious agent, other clinical and non-clinical factors such as disorders that suppress the innate and adaptive immunity, including cirrhosis, cancer and autoimmune diseases, genetic predisposition (e.g., polymorphisms in Toll-like receptor 4), host–pathogen interactions, reactivation of asymptomatic viral infections, underlying mechanisms of the acute illness, immune system responses, administered treatment, and the time-related direction of the underlying disease process progression [25,26].

This heterogeneity has a significant effect on clinical research outcomes. For example, this could be the case with a clinical trial, started for a breakthrough drug to treat sepsis or septic shock originated from a gram-negative microbe; according to the trial’s strict protocol, this pharmacological treatment will be only implemented for those critically ill patients admitted to the ICU within 4 h of the onset of sepsis/septic shock. Although in the beginning, the patient group to be recruited seems to be very specific, eventually patients are to be included with significant differences in innumerable biological characteristics, that may obscure the potential benefit of our study. For example, it is one case to treat a 19-year-old girl, who is an athlete and presented to the emergency department two hours earlier with meningococcal sepsis, and it is a very different case to treat a bedridden 80-year-old man with dementia, indwelling urinary catheter, Escherichia coli bacteremia and aspiration pneumonia complicated by acute respiratory distress syndrome (ARDS), or to treat a 60-year-old man with diverticulitis and intraabdominal abscess, but with no evident bacteremia.

### 2.2. Clinical Expression-Related Heterogeneity

Despite increased awareness and special focus on sepsis during recent decades, significant variability in sepsis diagnosis stills exists among critical care physicians. Even today, sepsis in its full manifestation cannot be determined with certainty, and if that sounds strange to the reader, the results of a study conducted some years ago emphasize this uncertainty: five case vignettes of suspected or confirmed infection and organ dysfunction were distributed to a sample of 94 intensivists practicing at academic institutions; the diagnoses assigned to each case presented a very wide variability, although ICU physicians are generally considered the specialists having the highest expertise in sepsis diagnosis and management [27]. The very identification of the septic patient in full clinical presentation sometimes proves to be difficult even for the experienced physician, as even laboratory evidence may be absent, and the diagnosis might rely only on clinical feeling. Its features are also confounded by comorbidities, and organ dysfunction may not be readily apparent [28]. Deterioration may be gradual and subtle or sudden. On the other hand, patient’s deterioration can be falsely attributed to sepsis in up to 20% of cases. [29]. In addition, no clear connection has yet been identified between the severity of sepsis and the number of organs failing. Although Sequential Organ Failure Assessment (SOFA) score has been linked to severity and prognosis in patients with sepsis [30], in the clinical setting a great heterogeneity in the type of organs affected is noted. For example, a patient with sepsis may be hemodynamically unstable, requiring two different vasopressors combined with corticosteroids, remaining with unaffected Glasgow Coma Scale and renal function, while other patients develop severe acute kidney injury together with severe neurological dysfunction while remaining hemodynamically stable. Regarding the impact of age, sepsis affects older patients unequally, and a significant percentage of them might already have an underlying organ dysfunction. To make matters worse, organ dysfunction may be in part caused by sepsis treatment rather than the sepsis itself [29].

### 2.3. The Definition of Sepsis as a Cause of Heterogeneity

In the 1970s, despite the use of antibiotics and the eradication of the pathogen, patients with sepsis continued to die. This led to the hypothesis that the underlying problem was not only the pathogen per se, but perhaps, to a greater degree, the patient’s inflammatory response, with physicians defending strongly in later years this kind of view regarding sepsis pathophysiology. In 1992, sepsis was defined as a clinical syndrome by the presence of both infection and a systemic inflammatory response syndrome (SIRS), defined by temperature, heart rate, tachypnea and white cell blood count [17]. These criteria were so general that practically any patient with a gastroenteritis or even a bad cold eventually met the definition of sepsis. It was the high mortality from sepsis that forced the scientific community to act in this way to make it easier for the physician to think of sepsis early and make a timely clinical diagnosis. However, the low specificity of the SIRS diagnostic criteria made the patient population meeting the diagnostic criteria for sepsis extremely large, causing enormous difficulties both in clinical practice and research. In 2001, a second definition of sepsis followed which was practically the same as the previous one, expanding mainly the list of clinical and paraclinical criteria and improving sensitivity at the expense of specificity; unfortunately, none of the criteria were specific for sepsis [18]. As many ICU physicians have already experienced in every day clinical practice, many critically ill patients with SIRS do not have sepsis and some septic patients do not have SIRS. Vincent et al. argued that SIRS is more sensitive but non-specific and that sepsis is not just the host’s response to an infection, but the host’s inflammatory response to infection leading to organ dysfunction, and emphasized that the response that predominates in the clinical phenotype varies across patients and over time in each patient [31]. Although in 2016 the third definition of sepsis (Sepsis-3 definition) followed and underlined that sepsis is life-threatening organ dysfunction from a dysregulated host response to infection, unfortunately it still remains very difficult to describe in detail this response, as no current clinical measure can accurately define what is meant by dysregulated host response and, in addition, pre-existing disease, comorbidities, medications, or interventions may alter the clinical and biological phenotype of sepsis [2]. Moreover, in the medical literature there are even objections and reactions to the current definition of sepsis [32], as the Sepsis-3 definition still relies on clinicians’ judgement of whether infection is present, as well as whether the organ dysfunction observed is really due to infection [27].

### 2.4. Sepsis and Precision (or Personalized) Medicine

What makes everyday reality complicated and problematic when dealing with patients with sepsis or septic shock is certainly this heterogeneity. The answer to how this large heterogeneous group of septic patients could be discriminated into smaller groups with distinct clinical characteristics might at the same time guide ICU physicians towards ‘precision medicine’, and for the most optimistic physicians ‘personalized’ medical treatment. Even though the terms ‘precision medicine’ and ‘personalized (or individualized) medicine’ are often used interchangeably, they are not identical. The focus of precision medicine is the identification of approaches that are expected to be effective for a specific group of patients based on their common characteristics. The ultimate goal is the ability to offer tailored care for each individual (personalized medicine), but predicting response to treatment at an individual patient level is still quite challenging, which is why, at present, efforts focus on precision medicine [33]. What is definitely well known is that sepsis still remains an enigmatic clinical syndrome, with complex pathophysiology incompletely understood, which can affect practically all organ systems, leading eventually to death in a very significant proportion of affected patients, though the organs involved and the degree of dysfunction may vary remarkably between patients [29,34]. The main challenge is not the determination of whether most patients who fulfill the generic sepsis criteria will receive any benefit from a specific therapeutic strategy, but the determination of the subgroup of patients that would benefit, as well as of correct timing during the disease process [1].

## 3. Critical Care Septic Patients

Critically ill patients can easily be characterized as the most heterogeneous population in the hospital, with the highest rates of multimorbidity, both acute and chronic. But septic critically ill patients hospitalized in the same ICU may well also have opposite outcomes despite receiving similar treatment [35].

In the past several years, new guidelines have emerged that mandate protocolized care in sepsis and septic shock patients. The Surviving Sepsis Campaign, a joint initiative of the Society of Critical Care Medicine (SCCM) and the European Society of Intensive Care Medicine (ESICM), currently represents the best available evidence for sepsis care, aiming to reduce mortality and morbidity from sepsis and septic shock worldwide. However, considerable criticism has been raised, arguing that not all patients should have the same management and the ‘one-size-fits-all’ approach should not be adopted [36,37,38]. Moreover, as already mentioned, most of the randomized controlled trials focusing on pharmacological therapies still fail to improve outcomes. For this reason, defining more precisely the clinical characters of heterogeneity in sepsis could be the first step in the effort to understand the potential response of different critical care patient groups to currently available therapeutic interventions. Despite the fact that the existence of heterogeneity in sepsis has been widely recognized and its presence in medical literature is strongly emphasized, until recently there were only very few studies attempting to group its characteristics. Thus, heterogeneity in sepsis seems to be an insurmountable obstacle in the quest to deliver improved treatment in septic critical care patients [23].

## 4. Phenotyping Sepsis in Critical Care Patients

What should be done? In order to identify specific groups of patients who could respond to specific interventions, the identification of clinical phenotypes is absolutely necessary [24]. The word ‘phenomenon’ derives from the Greek stem ‘φαίνομαι (phenome)’, a verb meaning ‘to appear’ or ‘to seem’. Johannsen in his 1909 definition of the ‘phenotype’ described it as an appearance-type characteristic [39]. Although, a patient’s phenotype is the sum of all expressed characteristics, i.e., the clinical expression, ‘phenotype’ could be also used to describe the trajectory and outcome of a condition, or for a clinical trialist to describe a set of presenting features implemented as criteria for enrolment into an experimental study [40]. Besides, sepsis is understood as a process, but only up to a point. For example, which genes are involved in the pathogenesis of sepsis? Gregor Mendel, the pioneer of modern genetics began with phenotypic traits and ended with genes. The answer to the question of how sepsis develops in each individual patient requires the solution of phenotypic puzzles, but phenotypes can also be the solution for guiding treatment [41]. By linking gene and phenotypic expression, novel mechanistic pathways or therapeutic targets for sepsis could be potentially discovered, and different biomarkers to predict the outcome of different septic patients, or even different gene-expression-based subclasses of sepsis that may require unique therapeutic approaches, could be discovered [42].

With all the aforementioned in mind, identifying the different phenotypes in septic patients seems an extremely difficult task as there is a lack of empirical evidence on how to individualize medical treatment of patients with sepsis based solely on clinical variables.

## 5. The Artificial Intelligence and Machine Learning Approach

In recent years, artificial intelligence and machine learning are being increasingly used in medicine. Artificial intelligence is a field that brings together expertise from disparate fields, including algorithms, statistics, information theory and cognitive science, teaching computers how to learn and act intelligently using data and performing complex tasks. One branch of artificial intelligence is machine learning; by accessing unsupervised large amounts of data and employing algorithms, machine learning mimics the way the human brain works, gradually and constantly improving accuracy [43]. The main idea is that unsupervised computers learn from the data they collect, usually based on electronic medical records, vital signs and/or laboratory results, and recognize patterns from large datasets; this type of approach has been proved to be superior to traditional analytic models and score models [44]. If artificial intelligence is considered as like the brain, then machine learning could be imagined as the way the mental process is completed by acquiring new cognitive abilities. Therefore, an algorithm can be created based on a very large amount of data, which finally leads to the creation of clusters (or phenotypes).

Clustering analysis is the type of unsupervised machine learning that has been recruited to investigate and identify different types of disease expression among different patient groups in multiple diseases, including cancer and asthma. Specifically, clustering analysis, by using advanced mathematical algorithms based on multiple specified variables, can quantify the similarity between individuals within a patient population. Eventually, by this approach, distinct novel subgroups can be generated, and these novel subgroups, which are not based on any a priori hypotheses, are the phenotypes [35,45].

Despite these promising results, it should be kept in mind that there are some limitations to consider when recruiting artificial intelligence techniques. For example, the optimal number of clusters that can be derived from any given dataset frequently cannot be identified by artificial intelligence itself. In addition, clusters derived from datasets determined with older sepsis criteria might differ significantly compared to when using current sepsis criteria. Moreover, machine learning techniques might perpetuate biases present in training datasets; thus, phenotypes derived should be internally and externally evaluated and clinical plausibility should be applied. If there is no separate cohort utilized for external validation, it might be hard to decide if given clusters are ‘artifacts’ or ‘real’, as clustering methods can find potential clusters even when natural clusters do not exist. Additionally, it might not be clear how to reconcile the different sets of patient subgroups identified by machine learning techniques and empirical approaches [46,47,48,49,50].

## 6. Temperature Phenotyping and Correlation with Immunological Profile

A basic distinct clinical trait used in septic patients is temperature. Although fever is often considered the main symptom of infection, some patients (10–20%) during sepsis remain hypothermic. From 2016 onwards, there are over 10 studies which found that hypothermia in the context of infection is associated with increased mortality, while fever was associated with decreased mortality (Table 1) [51,52,53,54,55,56,57,58,59,60,61,62,63].

Very recently, Baek et al. in a multicenter retrospective study using a machine learning approach in 15,574 patients with sepsis identified three different patient groups: hypothermic, normothermic, and hyperthermic, in which 90-day mortality rates were 27.4%, 19.6%, and 11.9%, respectively. Cluster analysis investigating age and body temperature (BT) demonstrated the following separate clusters of patients: ‘cluster A’ (>75 years and <36 °C), ‘cluster B’ (relatively younger age with wide range of BT), and ‘cluster C’ (relatively higher BT than cluster A). Each cluster had different 90-day mortality rates: 24.2%, 17.1%, and 17.0%, for cluster A, cluster B and cluster C, respectively. Mortality and BT had a negative correlation even in extreme ages, i.e., <75-year and ≥75-year age-groups [51]. Whether hypothermia’s effect on mortality is equal across all age groups is still under investigation.

This phenotypic dichotomy between hypothermia and fever and the relationship of hypothermia to mortality has been demonstrated and analyzed in recent large cohorts of patients, which distinguished extra phenotypic characteristics and associations.

For example, Thomas-Ruedell et al. in 6542 patients with sepsis confirmed that in septic patients a bimodal distribution of body temperature exists, with fever and hypothermia being the two main responses in human sepsis, with normothermia being a rather rare condition. Factors independently associated with hypothermia were community-acquired sepsis, abdominal infection, a lower BMI, higher age and lower environmental temperatures. On the contrary, factors independently associated with high fever were not only community- but also ICU-acquired sepsis, the presence of a pathogen in blood culture, and high procalcitonin values. In this study also, hypothermia in general was associated with higher mortality, but this association was reduced after adjustment for other risk factors [52].

Wu et al. studied 862 septic shock ICU patients and concluded that greater temperature burden below 36 °C or above 38 °C was associated with increased mortality in 21 days [53]. On the other hand, Shimazui et al. in a retrospective multicenter study using a sepsis cohort (1148 patients) and two validation cohorts of sepsis (1628 patients) concluded that, although in younger sepsis patients mortality was increased with hypothermia and decreased with fever, in elderly patients mortality was not associated with BT. These results might imply that younger and elderly septic patients demonstrate different inflammatory responses [54,61].

Bhavani et al. used group-based trajectory modeling to identify sub-phenotypes based on temperature trajectories. Group-based trajectory modeling is a specialized application of finite mixture modeling, an extension of cluster analysis, and is used to identify groups among patients who share similar trajectories with respect to a particular variable of interest [86,87]. Authors included 12,413 admissions in the development cohort and 19,053 in the validation cohort of patients admitted to the hospital from the emergency department with infection and receiving antibiotics within 24 h of presentation. They identified both in the development and validation cohort four temperature trajectory groups: ‘hyperthermic, slow resolvers’ (14.9%), ‘hyperthermic, fast resolvers’ (23.2%), ‘normothermic’ (32.8%) and ‘hypothermic’ (29.1%). Patients in the ‘hypothermic’ group were the oldest, had the most comorbidities and the lowest levels of inflammatory markers. On the other hand, the youngest patients with the fewest comorbidities and the highest levels of inflammatory markers fitted into the ‘hyperthermic, slow resolvers’ group. In the development cohort, ‘hypothermic’ patients had the highest in-hospital mortality rate (9.5%) while the ‘hyperthermic, fast resolvers’ had the lowest (2.9%). In the validation cohort, ‘hyperthermic, slow resolvers’ had the highest in-hospital mortality rate (10.2%), followed by ‘hypothermic’ 9.0%), while the ‘hyperthermic, fast resolvers’ again had the lowest (3.0%). Authors hypothesized that three distinct sub-phenotypes represented a hyperinflammatory (‘hyperthermic, slow resolvers’), a hypo-inflammatory (‘hypothermic’) and a well-balanced inflammatory sub-phenotype (‘hyperthermic, fast resolvers’) as indicated by its low mortality rate [55]. Although these sub-phenotypes and the lower mortality rates depicted are for patients with infection, i.e., a larger cohort that includes both septic and non-septic patients, they might still represent differential immunological responses.

Regarding the latter, the same group of researchers found in another study that, among two cohorts (120 patients with septic shock and 88 patients with Staphylococcus aureus bacteremia), patients could be classified again into one of the four previously validated temperature sub-phenotypes. Among patients with septic shock, the mortality rates were 51.7%, 36.8%, 29.6% and 22.2% for ‘hypothermic’, ‘hyperthermic, slow resolvers’, ’normothermic’ and ‘hyperthermic, fast resolvers’, respectively. Moreover, when plasma concentrations of 44 cytokines were compared, only for 4 cytokines (Granulocyte Colony-Stimulating Factor; G-CSF, chemokine ligand 2; CCL2, interleukins 7 & 10, IL-7 & IL-10) were plasma levels statistically different among the four sub-phenotypes; “hypothermic” patients exhibited the lowest mean values. Despite the finding that “hyperthermic, fast resolvers” had the most significant decreases in levels of five cytokines (interleukin 1 receptor antagonist; IL-1RA, interleukins 6 and 8; IL-6 & IL-8, G-CSF and Macrophage Colony-Stimulating Factor, M-CSF) over 24 h, whether any correlation between immunological profile and temperature trajectories exists still remains to be answered [62,88].

Interestingly, Bhavani et al. in two retrospective studies identified among COVID-19 patients four phenotypes similar to sepsis [60,64]. In particular, in their most recent study, they identified 5903 COVID-19 patients as ‘hyperthermic slow resolvers’ (25%), ‘hyperthermic fast resolvers’ (25%), ‘normo-thermics’ (36%), and ‘hypo-thermics’ (15%), with different clinical characteristics and mortality. Although it was found that the ‘hypothermic’ phenotype had marked coagulation disorders and the highest prevalence of venous thromboembolic events and cerebrovascular accidents, it was the ‘hyperthermic slow resolvers’ group who, in contrast with ‘hyperthermic fast resolvers’, had the highest levels of inflammatory markers, greatest hemodynamic instability requiring vasopressors, respiratory failure requiring mechanical ventilation and significantly higher mortality. Interestingly, the prevalence of the ‘hyperthermic slow resolvers’ phenotype for some reason drastically decreased over the course of the pandemic [64].

Harmon et al. obtained blood microarray data from 168 patients with sepsis within the first 24 h of their ICU admission. They observed that 30-day mortality was significantly increased in hypothermic patients (38% vs. 18%). This is in line with the aforementioned studies, but Harmon et al. also confirmed that patients with hypothermic or ‘cold’ sepsis had a unique gene expression profile providing a possible link to body temperature and early immunosuppression [89]. Confirmation of the above findings by future studies may suggest that hypothermic septic patients may benefit from immunostimulatory drugs, while those with fever may experience advantage from immunosuppressant treatment.

More and more researchers have started to investigate other aspects of the phenotypic relationship between temperature and mortality in septic patients. For example, Ito et al. in a prospective cohort study enrolling 1184 patients with sepsis admitted in 59 ICUs in Japan evaluated if there was any association between hypothermia and mortality of patients with sepsis according to body mass index (BMI). They concluded that septic patients with hypothermia and normal BMI may have a higher mortality risk than those with low or high BMI [63].

However, as other existing studies suggest that hypothermia in septic patients is independently associated with mortality, or even that the absence of fever is associated with higher mortality [56,57,59], and as a meta-analysis exists concluding that the presence of fever reduces mortality in septic patients as compared to normothermic subjects [58], the next step is to consider the application of therapeutic hyperthermia to patients with hypothermic or ‘cold’ sepsis [90]. To the best of our knowledge, there are two clinical trials in which rewarming of hypothermic patients with sepsis has been shown to be associated with a better prognosis [91,92].

## 7. Hemodynamic Phenotyping and Sepsis

Hemodynamics is the other clinical trait that may assist in phenotyping critically ill septic patients. Septic shock is defined by persistent hypotension requiring vasopressors to maintain a mean arterial pressure of 65 mm Hg and a serum lactate level greater than 2 mmol/L, despite adequate volume resuscitation [2]. Bearing in mind that systolic blood pressure (SBP) is routinely measured in the ICU, one could suggest the use of SBP in order to identify different phenotypes quickly and accurately.

Zhu et al. extracted data from a large medical database of 3034 septic patients admitted to an ICU, and after performing trajectory analysis they identified seven different SBP trajectories: in some patients (36.9%), SBP changed steadily but with not obviously increasing or declining trend (phenotype 1, mean SBP 100 mmHg); in phenotype 2 (7.5%), the SBP trend was stable (mean SBP 82 mmHg) and in phenotype 3 (8.4%) and phenotype 4 (21.3%) was generally stable, with SBP gradually increasing from 140 mmHg and from 110–120 mmHg to 120–130 mmHg, respectively. On the other hand, in phenotype 5 (15.3%) and phenotype 6 (8.2%), SBP was rapidly decreasing from about 130 mmHg to 100 mmHg and from about 150–160 mmHg to 110–120 mmHg, respectively, while in phenotype 7 (2.8%) SBP showed initially an increasing trend and then a decreasing one. The in-hospital mortality rates were 25.5%, 40.5%, 11.8%, 18.3%, 23.5%, 13.8%, and 10.5% for phenotypes 1 to 7, respectively. As in the multivariate analysis, phenotype 3 had the lowest mortality risk, and authors suggest that the SBP trajectory exhibited in phenotype 3 could be used as an ideal hemodynamic target for septic patients within 10 h of admission when aiming at improved outcomes. Besides, the fact that, when comparing phenotypes 2 and 6, the finding that phenotype 6 had a better prognosis suggests a rational proposal, i.e., that persistent hypotensive state has a worse prognosis compared to a large decrease in SBP. Therefore, by observing the SBP trend, clinicians could easily identify high-risk patients as early as possible and thereby provide timely treatment. It is worth mentioning that, in the same study, on a subgroup analysis, in phenotype 2 age was an independent predictor of in-hospital mortality: patients < 65 years had a higher mortality risk than patients > 65 years [65].

In another study involving 127 septic patients presenting in an emergency department, to whom non-invasive continuous hemodynamic monitoring (among others, cardiac index, CI and systemic vascular resistance index, SVRI) was applied, the application of a machine learning approach led to the identification of three different clusters with significantly different expression of hemodynamic parameters: phenotype 1 (56.7%) with high CI and normal SVRI, phenotype 2 (39.4%) with low CI and increased SVRI and phenotype 3 (3.9%) with very low CI and very high SVRI. Both phenotype 2 and 3 patients could benefit from patient tailored-fluid responsiveness directed therapies, while phenotype 3 patients could benefit more from vasodilator therapy. Interestingly, these three phenotypes differed significantly from each other in terms of 30-day mortality with most deaths (20%) occurring in phenotypes 2 and 3, while mortality was only 5.6% in phenotype 1 patients [93]. These findings are consistent with classical knowledge from several decades ago that septic patients, who are not able to increase their cardiac output, will develop metabolic acidosis and eventually will die. Although in 1981, Hess and colleagues proposed a model of sepsis in which the hemodynamic state was considered as a succession of different events [94]; it is worth mentioning that this approach is nowadays challenged, as current literature suggests that septic shock does not necessarily follow a linear natural history of clearly defined phenotypes succeeding one another; different authors suggest that sepsis and septic shock are completely different phenotypes, which appear independently of each other rather than consecutively and within a time continuum [66].

Recently, Geri et al. identified five distinct hemodynamic phenotypes in 360 septic shock patients based on clinical and echocardiographic parameters: ‘cluster 1’, the ‘well-resuscitated’ (16.9%), ‘cluster 2’, which had left ventricular (LV) systolic dysfunction (17.7%), ‘cluster 3’ characterized by LV hyperkinesia (23.3%), ‘cluster 4’ characterized by right ventricular failure (22.5%) and ‘cluster 5’ characterized by sustained hypovolemia (19.4%). ICU mortality in clusters 1, 2, 3, 4, and 5 was 21.3%, 50.0%, 23.8%, 42% and 38.6%, respectively [67].

It turns out that, by recognizing and differentiating early septic patients among different hemodynamic phenotypes, hemodynamic support, such as with vasopressors, could be somehow individualized and inotropes infusion and fluid resuscitation could be tailored to the need of each specific phenotype. Currently, there is at least one on-going clinical trial that investigates whether there is an association between the phenotype of septic cardiomyopathy, based on ultrasound characteristics, and the outcome of sepsis [95]. In addition, ANDROMEDA-2 study investigators seek to determine whether in early septic shock, i.e., <4 h of diagnosis, a peripheral perfusion-guided strategy compared to standard care, applied to different patient phenotypes identified by different clinical and hemodynamic criteria, could improve their outcome (mortality, duration of organ support and hospital length of stay) [96].

Bhavani et al., in order to develop and validate vitals trajectory phenotypes, applied group-based trajectory modeling to vital signs from the first 8 h of hospitalization in patients with suspected infection, both in training and validation cohorts (12473 and 8256 patients, respectively). By this approach, four phenotypes were eventually identified: ‘Group A’ (28%) with patients who were hyperthermic, tachycardic, tachypneic and with lower systolic and diastolic blood pressure; ‘Group B’ (13%), in which patients were also hyperthermic, tachycardic tachypneic (but not as much as in ‘Group A’) and hypertensive; ‘Group C’ (32%) and ‘Group D’ (27%) patients. Despite the fact that both ‘Group C’ and ‘Group D’ patients had lower body temperature, heart and respiratory rates, in ‘Group C’ patients were normotensive and in ‘Group D’ they had the lowest blood pressure. Regarding other special characteristics of the four phenotypic groups, it is worth mentioning that younger patients were in Groups A and B, while older patients were in Groups C and D, that ‘Group B’ had the highest prevalence of congestive heart failure, hypertension, diabetes mellitus, and chronic kidney disease, while ‘Group A’ had the lowest, and that, although Groups A and D had both the highest vasopressor use and the highest 30-day mortality, ‘Group D’ had a significantly lower risk of 30-day mortality when the administration of balanced crystalloids to saline was compared [97].

Apart from temperature and hemodynamic status, organ dysfunction can also be used for phenotyping septic critically ill patients.

## 8. Multiorgan Dysfunction Phenotyping during Sepsis

Probably the first study on multiorgan dysfunction and sepsis phenotyping was performed by Knox et al. in 2015. In this study, using machine learning techniques, 2533 septic patients admitted from the emergency department to the ICU were clustered in four distinct phenotypes; ‘cluster 1’ included patients with shock and elevated creatinine, ‘cluster 2’ patients with minimal multiorgan dysfunction syndrome, ‘cluster 3’ patients with shock with hypoxemia and altered mental status, and ‘cluster 4’ patients with hepatic disease. Mortality was 11%, 12%, 28%, and 21% for these clusters, respectively. Regression analysis showed that these phenotypic clusters were largely independent of age, cause of sepsis, obesity, and other comorbidities and that they differed significantly in the association between clinical outcomes and predictors, such as Acute Physiology And Chronic Health Evaluation (APACHE) II score [68].

Five years later, Ibrahim et al., using data from septic patients admitted to ICU (13728 records), calculating 63 vitals and laboratory tests collected within first 24 h of admission and following Knox’s machine learning methodology, also identified four clinically significant sepsis subpopulations with distinct organ dysfunction patterns representing either liver disease (phenotype 1) or cardiogenic dysfunction with elevated creatinine (phenotype 2) or minimal organ dysfunction (phenotype 3), or cardiogenic dysfunction with hypoxemia and altered mental status (phenotype 4). The populations identified were mostly independent of the origins of sepsis and 30-day mortality for these clusters was 28%, 55%, 25%, and 37%, respectively [69].

Zhang et al. in 2018 published the results of a retrospective study including 14,993 septic patients, in which by machine learning techniques four phenotypes were also identified: Phenotype 1 (69%) was considered the baseline type; phenotype 2 (9%) was characterized by respiratory dysfunction; phenotype 3 (11%) by multiple organ dysfunction (i.e., kidney, coagulation, liver, and shock), and phenotype 4 (11%) by neurological dysfunction. Phenotype 3 showed the highest mortality rate (45.4%), followed by phenotype 4 (27.4%), phenotype 2 (18.2%), and phenotype 1 (16.9%) [70].

Seymour et al., in a breakthrough study, analyzed retrospectively data from 63,858 septic patients who met Sepsis-3 criteria within 6 h of hospital presentation in three different cohorts using statistical, machine learning, and simulation tools. The investigators identified four sepsis phenotypes (labeled α, β, γ, and δ). Phenotypes ranged in size (from 13% to 33% of the cohort), each of them was distinct in terms of demographics, laboratory values and organ dysfunction patterns, and each of them was correlated with biomarkers and mortality. The ‘α phenotype’ (33%) had few abnormal laboratory values and less organ system dysfunction; ‘β phenotype’ patients (27%) were older, with increased comorbidity and kidney disease; ‘γ phenotype’ patients (27%) had increased levels of inflammatory markers, lower albumin levels and higher temperatures, whereas ‘δ phenotype’ patients (13%) had high serum lactate levels, elevated levels of transaminases and hypotension. The 28-day mortality rate for α, β, γ, and δ phenotype was 5%, 13%, 24% and 40%, respectively [71]. Although these four phenotypes had some relevance, with traditional severity of illness scores (APACHE, SOFA), it was clear that they represented distinct clinical groupings [98]. It is also worth mentioning that in the same study in simulation models the changes to the distributions among patient population for the α and δ phenotypes had substantial effects. This proved to be a very important finding as it suggested that whether a clinical trial will be effective seems to depend on the phenotype to which it will be applied, and this is certainly something we need to consider during the design of every new clinical trial [71,98].

This latter finding, i.e., that the outcome of RCTs might be affected by the proportion of participation for each phenotype group in the study population, highlights the study of Seymour et al. as a landmark and epoch-making study. It is rational to think that in order to conduct well-designed RCTs, it is very important to find and select the appropriate phenotypic population and deliver the appropriate therapeutic intervention at the most appropriate time. Even if a therapeutic intervention is truly beneficial for a proportion of the patient population, if it is not beneficial or even harmful for the remaining patients, then this intervention might be seen as ineffective overall. Based on the findings of Seymour et al., if we could recognize early in a septic patient a specific pattern of development of multiple organ failure, we could act prophylactically or therapeutically as physicians earlier in the patient’s favor, or in the case in which we want to conduct a clinical trial, we could apply selectively to the patient the intervention that will truly benefit him/her. It also seems that there is a great possibility for clinical studies that have already been conducted and have not shown, overall, any beneficial results, of repeating them in specific population groups with specific phenotypic characteristics, which may prove to be beneficial; this may be especially true for those trials which have shown benefit only in some specific subgroups [99].

Xu et al. investigated whether 72-h SOFA score trajectories can assist in phenotyping septic patients; a total of 4678, 3665, 12,282, and 4804 unique sepsis patients were recruited (one development and three validation cohorts), and four distinct clusters/phenotypes were identified: the ‘rapidly worsening’ (13.1%), in which SOFA score continuously increased from 4.5 at admission to more than 7 at 72 h; the ‘delayed worsening’ (20.5%), in which SOFA score decreased within the first 2 days from 5.2 to 3.7, and later increased over the last 24 h; the ‘rapidly improving‘ (41.3%), in which SOFA score continuously decreased from 5.54 to less than 3, and the ‘delayed improving’ phenotype (25.1%), in which SOFA score initially increased and later gradually decreased over 72 h. Each phenotype had different baseline characteristics. For example, those belonging to ‘rapidly worsening’ had greater comorbidity, acidosis, and visceral organ dysfunction, while ‘rapidly improving’ patients needed vasopressors but had no acidosis. Moreover, despite the fact that the ‘rapidly worsening’ phenotype had lower SOFA score (mean 4.5) compared to the ‘rapidly improving’ (mean 5.5), this phenotype had the highest in-hospital mortality (28.3% vs. 5.5%, respectively). The major finding of this study was that the authors were able to identify time-dependent progression motives that may be related to the differential response of specific organ dysfunction to standard of care interventions. As already previously suggested by the findings of Seymour et al., when conducting an RCT evaluating a therapeutic intervention to reduce the duration of organ dysfunction, if we recruited patients belonging to the ‘rapidly improving’ phenotype, the RCT would be unlikely to reveal any beneficial result [72].

Sharafoddini et al., based on demographics, vital signs, lab tests and organ dysfunction, also applied machine learning techniques among 5539 adult septic patients, identifying not four, but 12 phenotypes. The authors suggest that the methodology used could have paid closer attention to details when measuring similarities between patients. In-ICU mortality, in-hospital mortality and 30-day mortality rates varied significantly between clusters: mortality rates were lower in clusters 10, 11 and 8 and clusters 9 and 2 had the highest [73]. Reading their paper more thoroughly someone may suggest that 12 sub-phenotypes of five basic phenotypes were identified, highlighting the above-mentioned limitations of machine learning techniques, such as the lack of transparency and the relatively low reproducibility between studies at this time.

Five was also the number of phenotypes that Aldewereld et al. described in their study. The authors retrospectively applied machine learning techniques to a 1023-subject cohort with early septic shock. They characterized two ‘low-risk’ phenotypes (L), one ‘moderate-risk’ (M) and two ‘high-risk’ phenotypes (H). These were septic shock patients characterized as ‘fluid-responsive’ (phenotype L2), ‘fluid-refractory’ patients without multiorgan failure (phenotype L1), septic shock patients with respiratory failure (phenotype M), patients with multiorgan dysfunction (phenotype H1), and septic shock patients with hepatobiliary dysfunction and coagulopathy (phenotype H2). Phenotypes H1 and H2, although having different demographics, baseline illness severity scores, laboratory values and organ failure patterns, had similarly high mortality. Interestingly, this study provided unique evidence that different patient groups with different phenotypic traits, evolving differently in time, might eventually maintain the same signature of organ dysfunctions [74].

Ding et al. analyzed a cohort of 5782 septic patients with ICU hospitalization and derived three clinical phenotypes from a multivariate panel of physiological data using subgraph-augmented nonnegative matrix factorization. Frequent subgraph mining is a technique commonly used to identify patterns as subgraphs in a set of graphs based on a certain frequency threshold. This technique identifies frequent patterns in time series graphs, and has successfully phenotyped patient populations and predicted outcomes in multiple medical disorders. In this study, ‘phenotype 1’ patients were older, (73.1 years), overweight, male, and shared 17.0% 30-day and 12.0% in-hospital mortality. ‘Phenotype 2’ patients were younger (67.9 years), less overweight, male, with 28.4% 30-day and 24.8% in-hospital mortality, while ‘phenotype 3’ patients were the youngest (59.9 years), the least overweight, had 1:1 male-to-female ratio, and had the lowest mortality (10.1% 30-day and 7.3% in-hospital mortality). Once more, these phenotypes were characterized by distinct demographics and comorbidities, organ dysfunction patterns, and disease trajectories, with a clear association being evident between phenotypes and clinical outcome [75].

Papin et al. retrospectively analyzed 6046 patients with sepsis admitted to the ICU and also considered the loci of infection [76]. These investigators identified six phenotypic clusters: Phenotype 1 (40%) and 2 (4%) represented young patients without any comorbidities, admitted either for community-acquired pneumonia or meningitis/encephalitis respectively, ‘phenotype 3’ (6%) and ‘phenotype 4’ (27%) represented older adults either with chronic obstructive pulmonary disease, bronchial infection, and minor organ failures or increased comorbidities and organ failures respectively, ‘phenotype 5’ (15%) represented patients admitted postoperatively with a nosocomial infection, and ‘phenotype 6’ (8%) represented young patients with immunosuppression (e.g., AIDS, chronic steroid therapy, or hematological malignancy). Mortality at 28 days, 90 days, and one year was significantly different across different phenotypes, independently of baseline organ failures [76].

## 9. Sepsis Phenotypes, Fluid Status and Outcome

During sepsis and septic shock, proper fluid management remains a critical intervention aiming to maintain patient’s hemodynamic stability, organ perfusion, and ideal oxygen delivery. Fluid overload has a detrimental impact and applying precision medicine to septic critical care patients implies moving away from the well-known ‘standard-of-care’ fluid resuscitation approach that was widely applied over the past two decades. Currently, there is enough evidence to suggest that differences in fluid balance between different phenotypes is strongly associated with different outcomes and that identifying a subgroup of patients especially and particularly prone to fluid overload could be an essential step to optimize fluid management.

For example, Shald et al. among 320 septic patients admitted to ICU identified four phenotypes: ‘Phenotype 1’ (9.7%), included patients with multi-organ failure, ‘phenotype 2’ (15%) patients with respiratory dysfunction, ‘phenotype 3’ (18.1%) patients with neurologic dysfunction and ‘phenotype 4’ (57.2%) patients with other problems. Mortality rates between different phenotypes were also different; 48.4%, 20.8%, 39.7% and 13.7% for phenotype 1, 2, 3, and 4, respectively. Interestingly, fluid balances at 1, 2 and 3 days were also different, with phenotypes 1 and 3 having the largest volume balances at these time points [77].

Wang et al., in a prospective multi-center observational study enrolling 986 septic patients, investigated the effect of fluid balance latent trajectories on clinical outcomes, and detected three distinct trajectories/phenotypes: ‘Phenotype 1’ (6.5%), in which initially high fluid balance decreased quickly maintaining a lower level; ‘phenotype 2’ (85.3%), in which fluid balance was generally stable; and ‘phenotype 3’ (8.2%), in which fluid balance initially was high stable but later modestly decreased. When comparing phenotypes 2 and 3, ‘phenotype 3’ exhibited higher in-hospital mortality (71.6% vs. 46.9%), organ dysfunction (79.0% vs. 46.9%) and severe respiratory adverse events (58.0% vs. 29.7%), while ‘phenotype 1’ exhibited less adverse kidney events than ‘phenotype 2’ (47.4% vs. 51.6%) [78].

In the previously mentioned study of Zhang et al., each phenotype also showed different responses to fluid resuscitation; ‘phenotype 2’ received less fluid than all others, while ‘phenotype 3’ received the largest amount of fluid during the first 24 h. The impact of higher fluid balance in the first 48 h was disproportionately associated with outcome between different phenotypes: ‘phenotype 3’ had reduced risk of hospital mortality, and ‘phenotype 4’ increased [70].

Moreover, Ma et al., applying machine learning techniques among 1437 septic patients, tried to develop a sequential treatment rule regarding the ideal dose of fluid resuscitation and vasopressor use. Eventually, five phenotypes were identified: ‘Phenotype 1’ (baseline) included the majority of patients over all days; ‘phenotype 2’ included those patients with highest severity of illness; ‘phenotype 3’ included patients with renal dysfunction; ‘phenotype 4’ included those with respiratory failure; and ‘phenotype 5’ included patients with mild severity of illness, who also exhibited the lowest mortality rate (21%). The optimal fluid infusion followed the resuscitation/de-resuscitation phases with initial large volume infusion and late restricted volume infusion. While ‘phenotype 1’ transitioned to de-resuscitation phase on day 3, ‘phenotype 3’ transitioned on day 1 and, although phenotypes 1 and 3 seemed to benefit from early use of norepinephrine, ‘phenotype 2’ seemed to benefit from delayed use of norepinephrine. This finding confirms existing sepsis heterogeneity and makes clear one more time that an early treatment may be beneficial in the majority of septic shock patients, but delayed treatment may be advantageous for others [79].

## 10. Phenotyping ICU Trajectories: Mortality and Beyond

As has been already explained, sepsis itself seems to be an amalgam of multiple phenotypes, with different clinical presentation, different optimal management and a different outcome. Using machine learning techniques, we can also phenotypically investigate in detail and differentiate other aspects of critical care patients during ICU hospitalization.

For example, Zhang et al., in their retrospective multicenter cohort study with 22,868 septic patients admitted to an ICU, investigated when these were stabilized, when they transitioned to a state of persistent critical illness and whether such transition time varied between subclasses of these patients. They found that, although only 2.8% of the study patients developed persistent critical illness, these constituted 19% and 10% of total ICU and hospital bed-days, respectively, with elevated urea-to-creatinine ratio being a good biochemical signature of persistent critical illness. The authors identified five latent classes: Classes 1 (22.8%) and 2 (3.5%), in which SOFA score continuously increased and transition to persistent critical illness occurred at 16 and 27 days, respectively, and classes 3 (51.7%), 4 (11.2%) and 5 (10.8%), in which SOFA score continuously decreased and the transition to persistent critical illness occurred between 6 and 8 days. Both class 2 and class 5 patients exhibited high mortality rates (70% and 41.2%, respectively). Those patients who developed persistent critical illness also had overall higher mortality than those without persistent critical illness (25% vs. 16%), despite apparent stabilization with the development of persistent critical illness. The finding that those with decreasing SOFA score eventually developed persistent critical illness at an earlier stage than those with increasing SOFA score despite ICU treatment suggests that a septic patient’s response to the initial ICU treatment has a bearing on when they can be stabilized as having developed persistent critical illness [80].

Yang et al. carried out a retrospective analysis among 16,743 patients with sepsis and applying group-based trajectory modeling distinguished among those patients five separate trajectories of the SOFA scores at 72 h: in ‘group 1’ (32.8%), patients had the lowest SOFA score, which initially increased, then decreased and later increased over 72 h; in ‘group 2‘ (30.0%), patients had a higher SOFA score, which initially increased and later was stabilized; in ‘group 3’ (17.6%). patients had moderate SOFA scores (values between those in groups 1 and 2), which initially increased, then abruptly decreased within 72 h and finally continuously increased; and in ‘group 4’ (14.0%) and ‘group 5’ (5.7%), patients had relatively high SOFA scores with similar trends of change. Groups 2–5 had gradually increasing cumulative risk, while in ‘group 1’ risk remained low. It seems that early effective treatment was accompanied by decrease in SOFA score while inadequate infection control was accompanied by SOFA score stabilization. Higher risk of death was seen in those septic patients aged < 65 years and those who did not receive mechanical ventilation [81].

Soussi et al. again using machine learning techniques analyzed clinical and molecular data collected from patients in French and European ICU Registries; latent class analysis revealed two distinct phenotypes of sepsis-survivors among 467 patients: phenotypes A and B. ‘Phenotype B’ patients (48%) had more cardiovascular and kidney dysfunction, hematological problems and higher levels of inflammatory markers at ICU discharge, and significantly higher one-year mortality (34% vs. 16%). ‘Phenotype B’ was also independently associated with increased one-year mortality. It seems that, if we could recognize different groups of sepsis-survivors at ICU discharge, we could also recognize patients at higher risk of poor long-term outcomes and might manage to optimize their post-ICU treatment [82].

Similar were the findings of Taylor et al., who tried to investigate post-discharge mortality and rehospitalization rates among 20,745 septic patients. They used latent class analysis and they classified sepsis survivors into five distinct phenotypes: ‘Group 1’ (24.3%), i.e., patients with barriers to care (mortality 0.1%, readmission 8.6%); ‘Group 2’ (13.7%), patients who were previously healthy with severe illness and complex needs after discharge and barriers to care (mortality 2.1%, readmission 20.1%); ‘Group 3’ (27.6%), patients with multimorbidity (mortality 2.1%, readmission 26%); ‘Group 4’ (13.7%), patients with poor functional status (mortality 6.9%, readmission 21.6%); and ‘Group 5’ (20.7%), patients with severe illness and complex needs after discharge (mortality 7.9%, readmission 35.1%). The findings of this study strongly support the hypothesis that sepsis survivors constitute distinct clinical phenotypes with different needs for additional post-discharge support and indicate the need for predischarge classification, eventually aiming at a more individualized approach to post-sepsis care [83].

Boede et al., again using latent class analysis, explored depressive symptoms in 224 sepsis survivors for a follow-up period of 1 year and defined three different trajectories: one patient group (68%) exhibited a mild recovered trajectory, in which mild depressive symptoms recovered within 6 months, another patient group (12%) exhibited severe persistent trajectory, in which moderate-to-severe depressive symptoms persisted over the period of 1 year, and a third patient group (20%) exhibited severe recovered trajectory, in which severe depressive symptoms, although present 1 month after ICU discharge, finally fully recovered 6 months later [84].

Puthucheary et al., also using latent class analysis, assessed 159 sepsis survivors for a follow-up period of 2 years and identified two distinct groups: one group had a faster and more complete recovery (38.4%) and the other exhibited more persistent functional impairment (61.6%), with increased age, low educational level, and high comorbidity being independent risk factors for poor recovery. There was also a third group, which could not be classified into either trajectory, thereby illustrating the broad spectrum of sepsis outcomes among survivors [85].

## 11. Sepsis and Phenotypes: There Is More to Come

The number of studies on sepsis phenotyping continues to rise, taking into consideration the site of infection [100,101], the type of organ dysfunction, e.g., liver [102], kidney [103,104,105,106] lung [107,108] or coagulation [109,110,111,112], and the type of disease, e.g., COVID-19 [113,114,115,116,117,118,119,120,121,122,123,124,125,126,127,128] or non-COVID-19 ARDS [129,130,131,132,133,134,135,136,137,138,139,140,141,142,143,144,145,146,147,148,149,150,151,152,153]; they try to correlate phenotypes and associated harms of delayed time-to-antibiotics [154,155], and even to predict the response to treatment or the outcome [156,157,158,159]. Different phenotypes were also identified in septic children [160,161,162,163,164,165,166,167,168,169,170,171,172]. A complete survey of these topics is beyond the scope of this review and the reader is referred to the literature for further details.

## 12. Conclusions

**Figure 1:**
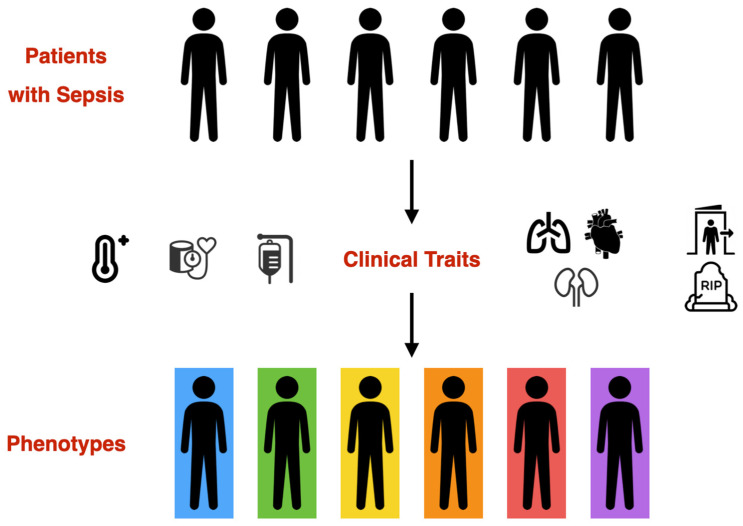
Sepsis phenotyping: clinical traits, such as temperature, hemodynamics, type of multi-organ dysfunction, fluid status and outcome can be used to identify distinct clinical phenotypes.

Sepsis has always been provided with a broad definition, in order to include the greatest number of patients at risk, at the cost of significant difficulties posed by heterogeneity for both clinical practice and research [173]. This considerable heterogeneity among patients and the adoption of a ‘one size fits all’ strategy in patient management can lead to widely divergent and even contradictory results [174]. For many years, no new therapies have become available and there is more than ever a need towards a personalized approach regarding sepsis care. Although currently it may not appear possible to incorporate phenotyping in our daily clinical practice and therefore offer personalized care to septic patients, small steps have already been made towards that direction as increasingly flexible and sophisticated clustering techniques allow analyses of big datasets and help identify distinct clinical phenotypes (Figure 1); different phenotypes may respond differently to treatment strategies. Defining more precisely the clinical characteristics of heterogeneity in sepsis could be the first step in our effort to understand the potential response of different critical care patient groups to currently available therapeutic interventions. The promise of precision medicine, therefore, is that a greater understanding of individual sepsis clinical expression, disease mechanisms, and heterogeneous treatment response patterns will lead to relocation from ‘poorly characterized’ to ‘personalized’ medicine and improved outcomes.

## Figures and Tables

**Table 1:** Main studies cited in the text regarding clinical phenotypes in sepsis.

Reference	Number of Patients	Number of Clinical Phenotypes
Temperature		
Baek et al. [51]	15,574	3
Thomas-Ruedell et al. [52]	6542	2
Bhavani et al. [55]	31,466 (2 cohorts)	4
Bhavani et al. [62]	208	4
Bhavani et al. [64]	5903	4
Hemodynamic status		
Zhu et al. [65]	3034	7
Daulasim et al. [66]	127	3
Geri et al. [67]	360	5
Ito et al. [63]	20,729 (2 cohorts)	4
Multiorgan dysfunction		
Knox et al. [68]	2533	4
Ibrahim et al. [69]	13,728 records	4
Zhang et al. [70]	14,993	4
Seymour et al. [71]	63,858 (3 cohorts)	4
Xu et al. [72]	25,429 (4 cohorts)	4
Sharafoddini et al. [73]	5539	12
Aldewereld et al. [74]	1023	5
Ding et al. [75]	5782	3
Papin et al. [76]	6046	6
Fluid responsiveness		
Shald et al. [77]	320	4
Wang et al. [78]	986	3
Zhang et al. [70]	14,993	3
Ma et al. [79]	1437	5
ICU trajectories		
Zhang et al. [80]	22,868	5
Yang et al. [81]	16,743	5
Soussi et al. [82]	467	2
Taylor et al. [83]	20,745	5
Boede et al. [84]	224	3
Puthucheary et al. [85]	159	2

## Data Availability

No new data were created in this study. All the data reported in this review were found in original articles cited in the text. Literature used to inform the text of this article was selected from PubMed.gov from the National Library of Medicine.
